# Exploring Modified Rice Straw Biochar as a Sustainable Solution for Simultaneous Cr(VI) and Pb(II) Removal from Wastewater: Characterization, Mechanism Insights, and Application Feasibility

**DOI:** 10.1021/acsomega.3c04271

**Published:** 2023-09-28

**Authors:** Yogeshwaran Venkatraman, Priya Arunkumar, Nadavala Siva Kumar, Ahmed I. Osman, Muruganandam Muthiah, Ahmed S. Al-Fatesh, Janardhan Reddy Koduru

**Affiliations:** † Department of Civil Engineering, Sri Krishna College of Engineering and Technology, Coimbatore 641008, India; ‡ Department of Chemical Engineering, KPR Institute of Engineering and Technology, Coimbatore 641047, India; § Project Prioritization, Monitoring & Evaluation and Knowledge Management Unit, ICAR Indian Institute of Soil & Water Conservation (ICAR-IISWC), Dehradun 248195, India; ∥ Department of Chemical Engineering, King Saud University, P.O. Box 800, Riyadh 11421, Saudi Arabia; ⊥ School of Chemistry and Chemical Engineering, Queen’s University Belfast, Belfast BT9 5AG, Northern Ireland U.K.; # Department of Environmental Engineering, Kwangwoon University, Seoul 01897, Republic of Korea

## Abstract

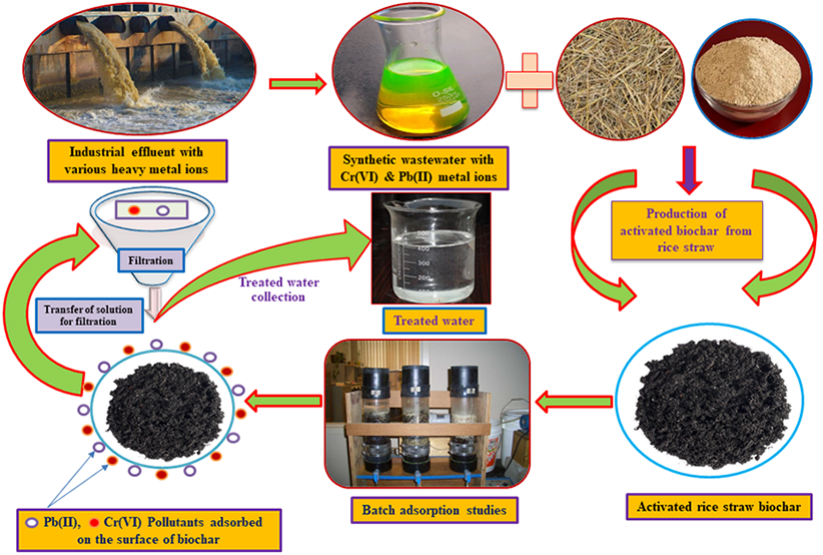

This study aimed to investigate the efficacy of a rice straw biosorbent in batch adsorption for the removal of chromium (Cr(VI)) and lead (Pb(II)) heavy-metal ions from wastewater. The biosorbent was chemically synthesized and activated by using concentrated sulfuric acid. The produced biosorbent was then characterized by using Fourier transform infrared (FTIR), scanning electron microscopy (SEM), energy-dispersive X-ray spectroscopy (EDX), and X-ray diffraction (XRD) analyses, which provided insights into surface morphology and functional groups. The study examined the effects of pH, rice straw dose, ion concentration, and contact time on metal ion adsorption. Optimal conditions for efficient removal (95.57% for Cr(VI) and 85.68% for Pb(II)) were achieved at a pH of 2.0, a biosorbent dose of 2 g/L, an initial concentration of 20 mg/L, and a contact time of 50 min in synthetic solutions. The isotherms and kinetics model fitting results found that both metal ion adsorption processes were multilayer on the hetero surface of rice straw biosorbent via rate diffusion kinetics. Thermodynamic investigations were conducted, and the results strongly indicate that the adsorption process is endothermic and spontaneous. Notably, the results indicated that the highest desorption rate was achieved by adding 0.3 N HCl to the system.

## 1 Introduction

Rapid industrialization and population growth have resulted in significant environmental challenges. The continuous discharge of harmful substances from industrial activities has led to widespread water pollution, rendering water unsuitable for drinking and other purposes. Furthermore, groundwater contamination by untreated wastewater has further deteriorated its quality. To enhance the surrounding environment’s quality, it is imperative to address and mitigate the detrimental impacts caused by these pollutants.^1^ Pollutants like heavy metals, dyes, and inorganic solvents are present in all major industrial effluents, which harms all living beings, even in very low concentrations.^2^ Heavy metals are highly poisonous among various pollutants, and nondegradable materials may create aqueous toxicity for surface water sources. Many heavy-metal ions are available, so chromium and lead ions play an important role in aqueous toxicity.^3^ Chromium and lead metals ions were produced by industrial activity such as tanning, electroplating, pulp and paper, fertilizers, etc.; these are all potential sources. Freshwater availability in the world is found to be exceptionally low, and there is a need for alternative ways to satisfy the requirement for freshwater.^4^ Hence, water remediation is the only solution to worldwide water scarcity.

Conventional water treatment methods were not used to effectively reduce the concentration of nondegradable pollutants.^5^ There is an urgent need for innovative treatment methods to control heavy-metal pollution, particularly in water. Also, the existing treatment technologies have disadvantages, like energy requirements, secondary sludge generation,^6^ incomplete pollutant removal, etc. Ion exchange, membrane separation, chemical precipitation, adsorption, electro-coagulation, etc. were used widely to remove the inorganic pollutants from the aqueous solutions.^7^ Among these methods, adsorption is one of the most widely used methods to remove nondegradable pollutants from wastewater. It refers to the accumulation of pollutants on the surface of an adsorbent through the attraction of van der Waals forces. Also, the generation of secondary sludge has been reduced considerably by adopting this method.^8^ Most adsorption can be done by adopting an organic material as an adsorbent. i.e., organically decomposable material such as fruit peels, tree bark, roots, seeds, fly ash, etc. The experimental study utilized rice straw adsorbent as charcoal activated with concentrated sulfuric acid. Then, the treated biosorbent was used to reduce the targeted pollutant concentrations from the synthetic solutions. Rice straw is an organic decomposable waste material produced by around 168.8 t annually in India. It has a large amount of cellulose (30–45%) and hemicellulose (20–25%) and a minimal amount of lignin (15–20%) organic components. These cellulose and hemicellulose provided the impact for huge biomass production, and lignin compounds increased the adsorption efficiency. Many research works have been conducted using rice straw adsorbent material because of its excellent organic structure and high fiber content.^9,10^ It is a ridiculously cheap and easily available substance that will be used as an organic biosorbent for this batch experimental study. Rice straw was selected as the adsorbent material for this experiment to test its performance in removing chromium and lead ion concentrations by batch adsorption techniques.

This research extensively discusses the adsorption mechanism for the removal of heavy metals from synthetic solutions through batch adsorption studies. The primary objective of this experimental study is to develop a cost-effective adsorbent material from rice straw to effectively remove heavy-metal contaminants from wastewater. The physical and chemical properties of the chemically activated rice straw adsorbent were thoroughly evaluated by using techniques such as Fourier transform infrared (FTIR), scanning electron microscopy (SEM), energy-dispersive X-ray spectroscopy (EDX), X-ray diffraction (XRD), and Brunauer–Emmett–Teller (BET) surface area analysis. The impact of various adsorption parameters, including pH, reaction time, dose, and concentration, was investigated under different conditions. The experimental data obtained from these studies were analyzed by using different isothermal and kinetic models. The thermodynamic analysis provided insights into the nature of adsorption, while desorption and regeneration studies demonstrated the maximum recovery of the spent adsorbent. Also, the pollutants adsorbed by the rice straw biochar material were disposed of through landfilling in a remote area to avoid the problem of creation due to those toxic substances.

## 2 Materials and Methods

### 2.1 Preparation of Biosorbent and Stock Solution

Rice straw material was collected in the agricultural fields of the Coimbatore area in India and cut into small pieces, then dried for 8 h in sunlight to evaporate the water molecules. Then, the sun-dried material was ground using the mechanical crushing machine and sieved at different rates. The retained fractions of sieves in different sizes of 300 to 600 μm, 75 to 150 μm, and <75 μm were used, and the collected rice straw from the sieves was completely washed using distilled water to remove the impurities. The experimental study used a standard 75–150 μm charcoal adsorbent material. The rice straw material was kept in an oven after washing, allowing further heating for 24 h at 100 °C. The concentrated sulfuric acid was added after the sample was activated from an oven and kept for 2 h. After that, the samples were washed with double-distilled water to remove the acidic nature of biochar. Finally, the samples were taken from an oven and placed in the desiccator for further studies. Potassium dichromate (K_2_Cr_2_O_7_) and lead sulfate (PbSO_4_) were used to prepare the stock solution for Cr(VI) and Pb(II) ion adsorption in double-distilled water. Here, 1000 mg/L distilled water was added with 100 mg of potassium dichromate and lead sulfate powder, and the stock solution was prepared. All of the chemicals used for this study were purchased in analytical grade, and double-distilled water was used for dilution.

### 2.2 Batch Adsorption Study

Through batch adsorption technique, the experimental analysis was carried out to check the performance of prepared rice straw adsorbent in various operating parameters. The prepared synthetic solution (100 mL) of Cr(VI) and Pb(II) was taken in a conical flask, and the pH alterations were made by adding 0.1 M of NaOH or 0.1 M of HNO_3_. The specified level of the adsorbent dose was measured and added to the synthesis solution for experimental analysis. Using the orbital shaker, the solution was mixed well with a rotation speed of 150 rpm for 1 h. As per the standards for separating the solution and rice straw adsorbent, the final pH of the solution was determined using a pH electrode at room temperature (25 °C) and filtered through cellulose acetate filter paper (0.45 μm). Using atomic adsorption spectroscopy (AAS), the initial (*C*_i_) and final concentrations of metal ions in the synthetic solution and the equilibrium concentration of metal ions (*C*_e_) were obtained.

### 2.3 Effect of pH on Ion Removal and pH_ZPC_

To find out the capacity of adsorption using rice straw biochar, the adsorbent was placed into a solution containing metal ions (Cr and Pb). The aqueous solution pH was altered by adding HNO_3_ and mixed well for up to 3 h for the equilibrium attainment. For this experimental analysis, 0.5 g of rice straw adsorbent samples were used, with an average particle size ranging between 75 to 150 μm. The pH_ZPC_ characterization surface property studies evaluated the net electrical neutrality of rice straw adsorbent’s net electrical neutrality. The pH_ZPC_ of the rice straw adsorbent was examined using the pH drift method. With a known pH of 0.01 mol/L NaOH, the rice straw adsorbent of 25 mg was mixed, and the nitrogen gas was allowed to the suspension for 2 h. Under the nitrogen gas involvement, the suspension of deoxygenized sample and its pH was adjusted from 2.0 to 7.0 with continuous stirring for 48 h. The initial and final pH of the solution was obtained and plotted to identify the pH_ZPC_ when pH_Initial_ = pH_Final_.

### 2.4 Effect of Contact Time

For this study, 0.5 g of rice straw adsorbent sample with an average size of 75–150 μm was added to the synthetic solution with an optimum pH level. Varying the time interval from 10 min to 2 h, the impact of adsorption efficiency has been investigated at room temperature.

### 2.5 Effect of Adsorbent Dose

The effect of adsorption efficiency was examined by altering the dose of rice straw biosorbent from 0.5 to 3.0 g/L, while keeping the optimum pH and contact time values obtained from the previous experimental analysis constant. Under room temperature, the experimental analysis was performed with 50 mg/L initial metal ion concentrations.

### 2.6 Effect of Ion Concentrations

In this study, the effect of different initial concentrations of metal ions (ranging from 20 to 100 mg/L) on the efficiency of metal ion adsorption was investigated at room temperature. The optimum pH, rice straw dose, and contact time obtained from previous experimental studies were used in this investigation. Based on the above variations in adsorption parameters, the targeted metal ions and their adsorption by the rice straw adsorbent have been calculated using eq 1 in equilibrium time.
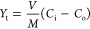
1

The volume and mass in the adsorption system were denoted by *V* (L) and *M* (g), and *C*_i_ and *C*_o_ (mg/L), respectively, represent the initial and final concentrations of metal ions in the synthetic solution. The system of mass balance approach for metal ion adsorption was calculated using eq 2.
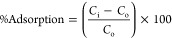
2

### 2.7 Rice Straw Adsorbent Characterization

The surface area of rice straw biochar adsorbent was obtained by nitrogen adsorption–desorption, conducted at −196 °C. The rice straw biochar was kept in the furnace for 3 h at 250 °C, and the gas molecules were eliminated. Further, the BET surface area analysis determined the rice straw biochar’s vacuum area. With the help of eq 3, the meso- and micropores availability and their behavior were obtained, and the size of particles was calculated by the Dubinin–Radushkevich (D–R) process.
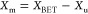
3

*X*_m_ and *X*_u_ represent the average meso- and micropores, respectively, and *X*_BET_ represents the surface area of the rice straw biochar adsorbent. The quantity of nitrogen required for surface area analysis was calculated by taking pressure (*P*/*P*_o_ ∼ 0.99), and eq 4 may be used to determine the pore volume (*S*_V_) in the adsorbent.
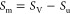
4

Here, *S*_m_ represents the average micropore and *S*_u_ represents the average mesopore of the rice straw biosorbent. Also, the rice straw adsorbent’s pore diameter (*S*_P_) can be obtained by using eq 5.
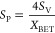
5

Equations 3 and 4 are called Dubinin–Radushkevich (D–R) process and Barrett–Joyner–Halenda (BJH) model, respectively. A few characteristic studies were conducted on the prepared biosorbent and its characterization. To check the functional groups, FTIR analysis was performed. The solution’s pH was maintained at 5.0; Cr(VI) and Pb(II) concentration of 50 mg/L with 1 g/L rice straw biochar adsorbent was used. After 3 h of shaking to attain the equilibrium time and 15 min of ideal conditioning, the final suspension was subjected to FTIR analysis with a bandwidth of 400 to 4000 c^–1^. Rice straw biochar adsorbent and its surface morphology were tested by SEM analysis with 50 μm of distance and 20 kV of power. The presence of targeted ions and their accumulation level has been obtained through EDX analysis. The crystalline structure of the rice straw biosorbent’s surface was examined by X-ray Diffraction (XRD) analysis at various peak intervals. The sizes and phases of crystalline structure that exist in the biosorbent were identified using the XRD instrument with CuK-α radiation. The X-ray diffraction (XRD) analysis was conducted using 40 kV and 250 mA of power to obtain the peaks, which were then compared to the Joint Committee on Powder Diffraction Standards (JCPDS) standards using the reference code of -00-002-1035.^11^

The Zero Point Change (ZPC) of pH was evaluated by varying the pH of the metal ion solution from 2 to 11 with the addition of 0.01 M NaCl as the electrode base. To determine the point of zero charge (pH_zpc_), 0.5 g of rice straw biochar was separately added to 25 mL of different as-prepared solutions with varying pH values. The mixtures were then shaken at room temperature for 24 h. Afterward, the supernatant was carefully decanted, and the pH values of the supernatant were measured. By plotting a graph of the initial solution pH values against the supernatant pH values, we determined the pHzpc value was determined. The final pH (pH_f_) of the solution was determined and recorded precisely. The difference between the initial pH (pH_i_) and the final pH (pH_f_), denoted as ΔpH = pH_i_ – pH_f_, was calculated. The calculated ΔpH values were then plotted against the initial pH (pH_i_) of the solution. The intersection point of the *x*-axis in the graph, where ΔpH equals zero, represents the point of zero charge (pH_pzc_).

Isothermal and kinetic studies have evaluated the nature and process of metal ion adsorption using the rice straw adsorbent material. The interactions between solid and liquid phase changes of adsorbent material were examined through isotherm and kinetic studies. 25 mg of Cr(VI)- and Pb(II)-loaded rice straw biosorbent was taken for reusability studies. Before reusing, the rice straw biosorbent was agitated in 50 mL of HCl solution for 1 h and regenerated. The adsorbent was rinsed twice in the distilled water to desorb the adsorbed metal ions. Desorption studies were conducted to recover the spent adsorbate from the aqueous solutions using concentrated hydrochloric acid with a normality range of 0.1 to 0.4.

### 2.8 Isothermal Studies

The system and capacity of adsorption may be assessed using isotherm studies under controlled temperature, adsorbent dose, and ion concentrations. The capacity of adsorption (*q*_max_) and the concentration of equilibrium (*C*_e_) were related through isothermal studies to select the most suitable adsorbent material. Also, various isothermal studies have analyzed the interaction between the adsorbent material and targeted pollutants. Among the various isotherm studies, Langmuir and Freundlich have been the most popular and convenient methods to determine the nature of metal ion adsorption.

The Langmuir isotherm study helps to determine the behavior of solid and fluid phases by transferring the gas and solid phases. This model follows a homogeneous type with a monolayer process of adsorption, and it assumes the process of pollutant uptake by the adsorbent occurred through chemical reactions only.^5^ Based on the assumptions, the equation of the Langmuir model was developed and expressed as
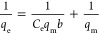
6where *q*_e_ represents the amount of adsorbate/mass of adsorbent in equilibrium (mg/g), *q*_m_ represents adsorption capacity in monolayer (mg/g), and *b* represents the constant of the Langmuir model related to the binding energy.

The Freundlich isotherm model is also a type of isothermal study that helps to prevent the multilayer adsorption process by its heterogeneous nature. The gas adsorbed by the adsorbent and its variations was examined through this model, and this assumption was used to form the Freundlich isotherm model equation expressed as,
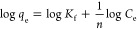
7

The Freundlich capacity constant is represented by *K*_f_, which is the indicator to determine if the equilibrium concentration reached a normal level. Also, the parameter 1/*n* represents the Freundlich intensity based on the heterogeneous adsorption system. When the adsorption process follows both Langmuir and Freundlich studies, the experimental data must follow with either Langmuir/Freundlich fit. The other isothermal models may fit these cases’ expected and obtained experimental data. Sips, Toth, and Fritz–Schlunder’s isothermal models were used widely to check the fitting of pollutant adsorption through the Langmuir/Freundlich model. These studies help to identify the monolayer/multilayer-type adsorption with a homogeneous/heterogeneous nature.

The sips isothermal model was evaluated to check the process of metal ion adsorption. Equation 8 is a mathematical expression of the Sips model, which is a combination of the Langmuir and Freundlich isotherms. It can predict the existence of heterogeneous sites at the limiting behavior levels of adsorption.^12^ The Sips isotherm assumes a monolayer adsorption process and neglects the adsorbate concentrations.
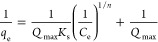
8

Here, *Q*_max_ represents the adsorption capacity and *K*_s_ represents the equilibrium constant. The factor of heterogeneity is denoted by *n*.

The Toth isotherm model was developed to address the inconsistencies between the experimental and equilibrium data observed with the Langmuir isotherm model. The Toth isotherm model explains the adsorption process at both low and high concentrations of metal ions. The Toth isotherm was represented in mathematical form by referring to eq 9.
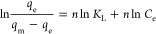
9

Here, *n* represents the Toth model exponent, *K*_L_ is the Toth model constant, and *C*_e_ denotes the equilibrium concentration of the adsorbate. This equation describes the relationship between these parameters in the Toth isotherm model.

The Fritz–Schlunder isotherm model, also known as the four-parameter model, is employed to analyze experimental data over a broad range and improve the adsorption process through empirical equations.^13^ By utilizing nonlinear regression analysis, the parameters of this model can be determined. Equaiton 10 represents the Fritz–Schlunder isotherm model.
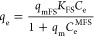
10Here, *q*_mFS_ represents the adsorption capacity in the maximum range, *K*_FS_ represents the equilibrium constant, and MFS is called the exponent model.

### 2.9 Kinetic Studies

The relationship between solid–liquid phase and retention rate has been analyzed through the linear mode of study by kinetic studies using various kinetic models. The optimum values of adsorption parameters were considered from batch studies, and the curve was developed to check the linearity between adsorbent and adsorbate concerning the physical/chemical mode of adsorption.^14^

The pseudo-first-order model assumes the solute change rate is proportional to the concentration of saturation in the adsorption system, and the pollutant uptake by the adsorbent was obtained only through solid–liquid adsorption systems.^15^ The pseudo-first-order linear equation was developed and expressed in eq 11 by referring to the above-said assumptions.
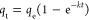
11

The pseudo-second-order model assumes that the pollutant adsorption by the biochar adsorbent is directly proportional to the number of active sites available in the biochar material.^16^ The equation of this kinetic model is expressed in eq 12 based on the above assumption.
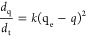
12

The performance of the biochar adsorbent was assessed in the presence of gas molecules during the initial phase of the metal ion uptake process. The observation made was that as the desorption rate decreased, the amount of metal ions adsorbed from the solute increased exponentially.^17^ Using this assumption, the Elovich kinetic study can be represented by the linear equation shown in eq 13.
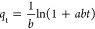
13

The rate-controlling step of heavy-metal adsorption by the biosorbent was evaluated by using the Boyd kinetic model and its data. The Boyd kinetic equation can be expressed in eq 14. The value of *D*_i_ can be calculated using eq 15 after the attainment of *B* values calculated from the Boyd kinetic plots.
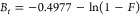
14
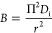
15

Intraparticle diffusion (IPD) should be applied only in the higher solute concentrations in the batch adsorber. The origin passage of the plot of *q*_t_ versus *t*^1/2^ indicates that intraparticle diffusion controls the adsorption process. On the other hand, if the data shows a multilinear curve, it indicates that two or more steps are involved in the adsorption process.^18^Equation 16 expresses the mathematical form of IPD studies.
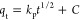
16

## 3 Results and Discussion

### 3.1 Adsorbent Characterization

#### 3.1.1 Pore Size and BET Area Analysis

**Figure 1:**
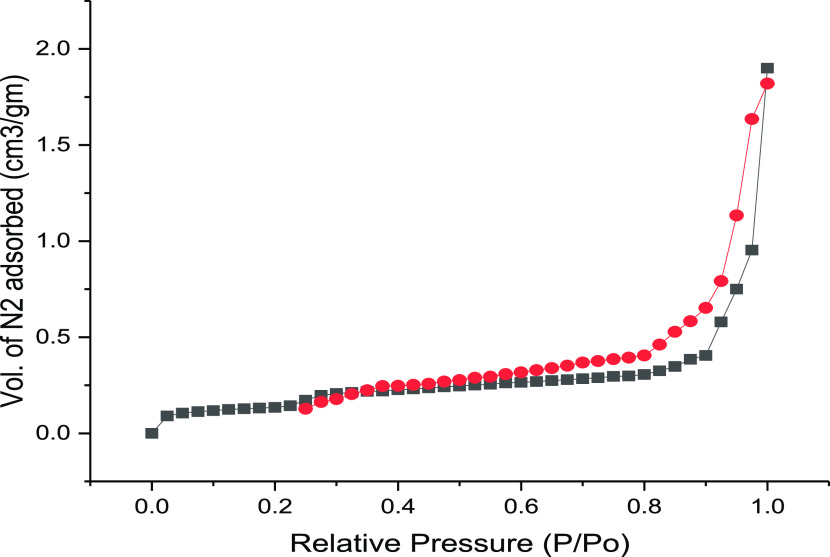
BET surface area analysis by nitrogen adsorption and desorption.

The rice straw biosorbent’s micro- and mesopore size and surface area have been evaluated by BET surface area analysis, as shown in Figure 1. The values of meso- and micropores and their levels were obtained from Figure 1 and are represented in Table 1. Referring to Figure 1, the rice straw biosorbent follows the type II category. i.e., the adsorbent has both meso- and micropores on the surface.^19^ Micropores were observed in the first curve, and the mesopores’ presence was observed in the second curve concerning the relative pressure shown in Figure 1. The commercial activated carbon’s surface area was greater than the rice straw adsorbent’s surface area (534 m^2^/g) with a pore volume of 0.254 cm^3^/g. The BET surface area of rice straw biosorbent was found to be very low compared to other commercial activated carbons like fox nut −2636 m^2^/g,^20^ hazelnut shells −717.738 m^2^/g,^21^ and Wooden chips of spruce and birch −530 and 647 m^2^/g,^22^ respectively. Hence, the surface area of the prepared adsorbent material has enough space to accumulate pollutants from the sources.

**Table 1:** Pore Properties of Rice Straw Adsorbent Material

s. no.	type of property	calculated value
1.	surface area	534 m^2^/g
2.	pore volume	0.254 cm^3^/g
3.	pore radius	75.934 Å
4.	surface area (BET)	27.384 m^2^/g
5.	average pore diameter	0.14 nm

#### 3.1.2 FTIR Studies

**Figure 2:**
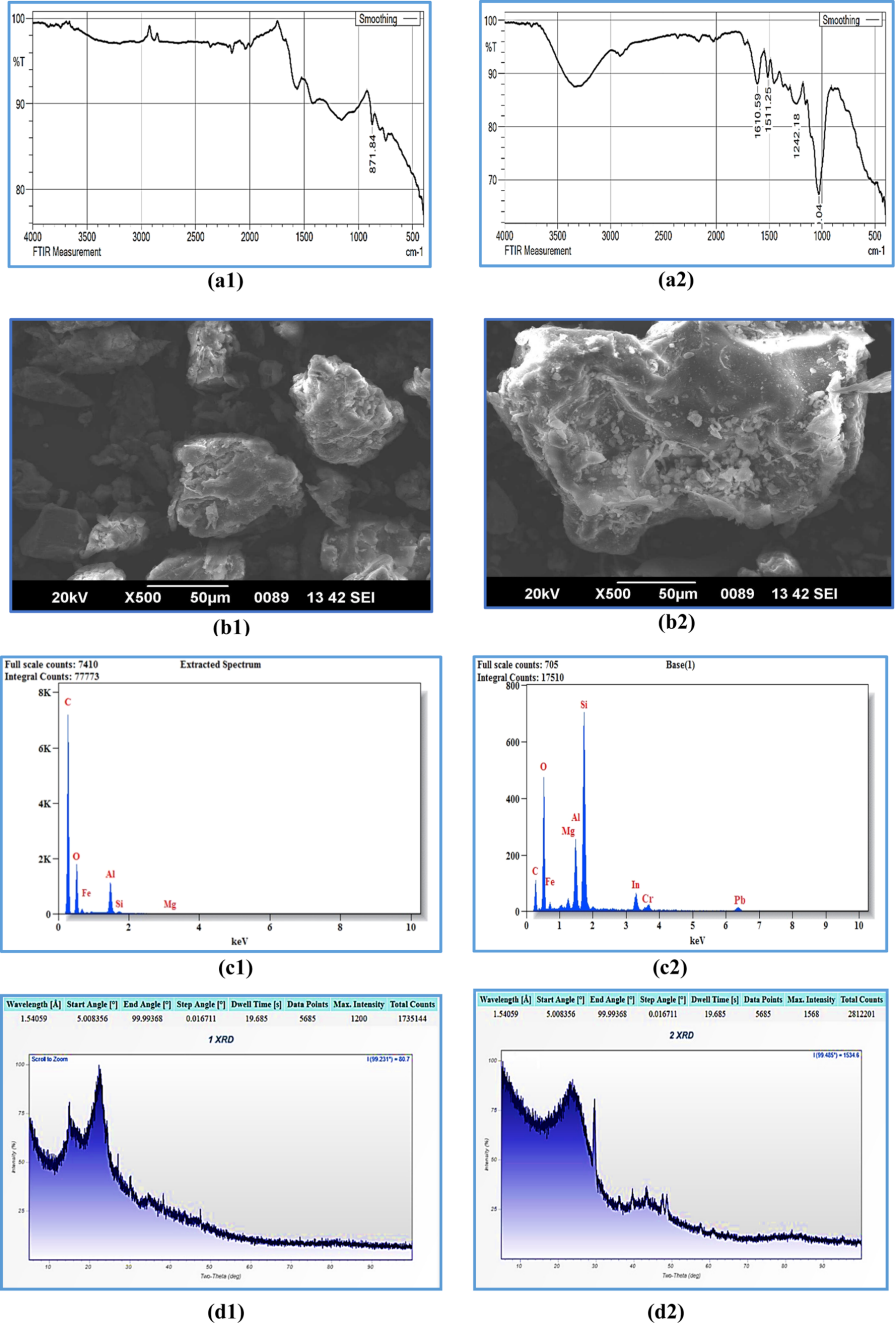
(a1, a2) FTIR peaks, (b1, b2) SEM image, (c1, c2) EDX analysis, and (d1, d2) XRD peaks of rice straw biosorbent before and after metal ion adsorption, respectively.

FTIR analysis was conducted to identify and determine the various functional groups present in the rice straw biochar adsorbent pre and postadsorption process. Figure 2a1,a2 shows the untreated and treated biosorbent’s spectra, and the results were compared with each other for the metal ion adsorption process. In this, the peak in the range from 3200 to 3550 cm^–1^ represents the N–H amino bond groups with the presence of hydroxyl groups. The concentration of the functional groups was linked with each band, and it is similar to the difference between the intensity of bands. At a frequency level of 2860 to 3420 cm^–1^, the hydroxyl groups and their presence were observed. The two peaks were observed at 2295 and 2852 cm^–1^, indicating the stretching vibrations of the methylene hydrogen and asymmetric and symmetric −CH functional groups. The C=N amides or ketones were attributed to the aromatic stretching of C=C and C=O was observed at an intense peak of 1610 cm^–1^. Various peaks were observed at 1400, 1053, and 1034 cm^–1^ due to the presence of C–H asymmetric bends, and it describes the stretching of alcohol, sulfoxides, carbohydrates, or polysaccharides-like substances. The peak at 1050 cm^–1^ indicates the broad band in medium intensity level, and it was assigned to υ (C–O–C) asymmetrical stretching. The variations between functional groups and their concentrations with band association are similar to the variations in band intensity.^23^

Compared to the untreated rice straw biosorbent, the treated adsorbent’s spectra were shifted to a small range, which was observed in Figure 2a2. The shifts observed in the biomass were due to the binding action of Cr and Pb metal ions with amino and hydroxyl groups. The changes in the bands observed in the FTIR analysis confirm that the rice straw biosorbent has undergone a process of metal ion uptake. The intensity from 3000 to 3800 cm^–1^ represents the stretching of hydroxyl (−OH) compounds, and the medium range of peaks from 1400 to 1600 cm^–1^ represents the stretching of carbonyl (−C=O) groups. The hydroxyl groups and their involvement with the binding of Cr and Pb ions were confirmed with the biosorbent by referring to minor shifts in peak frequency. The main functional groups of carbonyl, hydroxyl, amide, sulfonate, carboxyl, and phosphonate were identified, and these are all responsible for the biosorption process.^24^ The rice straw biosorbent possesses functional groups that enable it to adsorb heavy-metal ions from aqueous solutions.

#### 3.1.3 SEM Analysis

The changes in the surface of the rice straw biosorbent due to pollutant adsorption were examined using SEM, as presented in Figure 2b1,b2, which shows images taken before and after the process. This figure shows the raw adsorbent material surface before taking the pollutant from the sources. It was seen that uneven pores and their presence on the rice straw biochar’s surface in the SEM images and these pores might help to receive the pollutants from the sources. Also, the surface was rough and used to keep the pollutants on the adsorbent.^25^ The second SEM image (Figure 2b1) shows the adsorbent surface after the pollutants are removed from the sources. The pores in the adsorbent surface were filled with pollutants, and the flat surface was observed after the saturation point. The pollutants were occupied in the pores’ inner walls due to van der Wall’s force attraction and reached equilibrium.^26^ These SEM images confirmed that the process of pollutant adsorption happened on the adsorbent’s surface.

#### 3.1.4 EDX Analysis

The surface morphology of the prepared rice straw biochar adsorbent has been examined by EDX analysis to confirm the presence of targeted pollutants of Cr(VI) and Pb(II). Figure 2c1 shows the biosorbent before the adsorption process, while Figure 2c2 shows the biosorbent after it has taken up the targeted metal ions. The peak was developed due to calcium, carbon, and oxygen contents in the first figure. After adsorbing the pollutants, many peaks were generated and represented in the second figure. Many functional elements, such as magnesium, aluminum, silica, and iron, are seen in that figure, along with the targeted metal ions of chromium and lead. Hence, it confirms the ability of pollutant uptake for the adsorbent material. The adsorbent material was treated with concentrated sulfuric acid, and this acid can react with the hydroxyl groups. Due to this reason, the nonionic functional elements of silica, alumina, etc., were deposited on the adsorbent’s surface and created a complex nature.^27^ It is difficult to destroy the complex ions in the adsorbent due to the protonation of charged ions in the raw material.^28^

#### 3.1.5 XRD Analysis

Figure 4a,b shows the XRD peaks obtained for the raw adsorbent and activated biochar adsorbent, respectively. The charcoal adsorbent had a higher intensity and a more defined crystalline structure compared to the raw adsorbent. The rice straw biochar had diffraction peaks at 150, 230, 270, 310, 385, and 470 at 2θ, matching with 70, 100, 50, 40, and 10 *jkl* planes. Based on the high intensity and nature of the diffraction points, it was confirmed that the adsorbent material possessed a crystalline nature. Figure 2d1,d2 shows the XRD peaks before and after the taking up of metal ions by the adsorbent.

#### 3.1.6 Batch Adsorption Studies

The batch adsorption process has evaluated the optimum adsorption parameters and values in various operating conditions. Altering the solution’s pH, rice straw biosorbent dose, the concentration of ions in the wastewater, and contact time between adsorbent and adsorbate, the effect of adsorption efficiency by the biosorbent was examined using the following experimental studies.

### 3.2 Effect of pH

**Figure 3:**
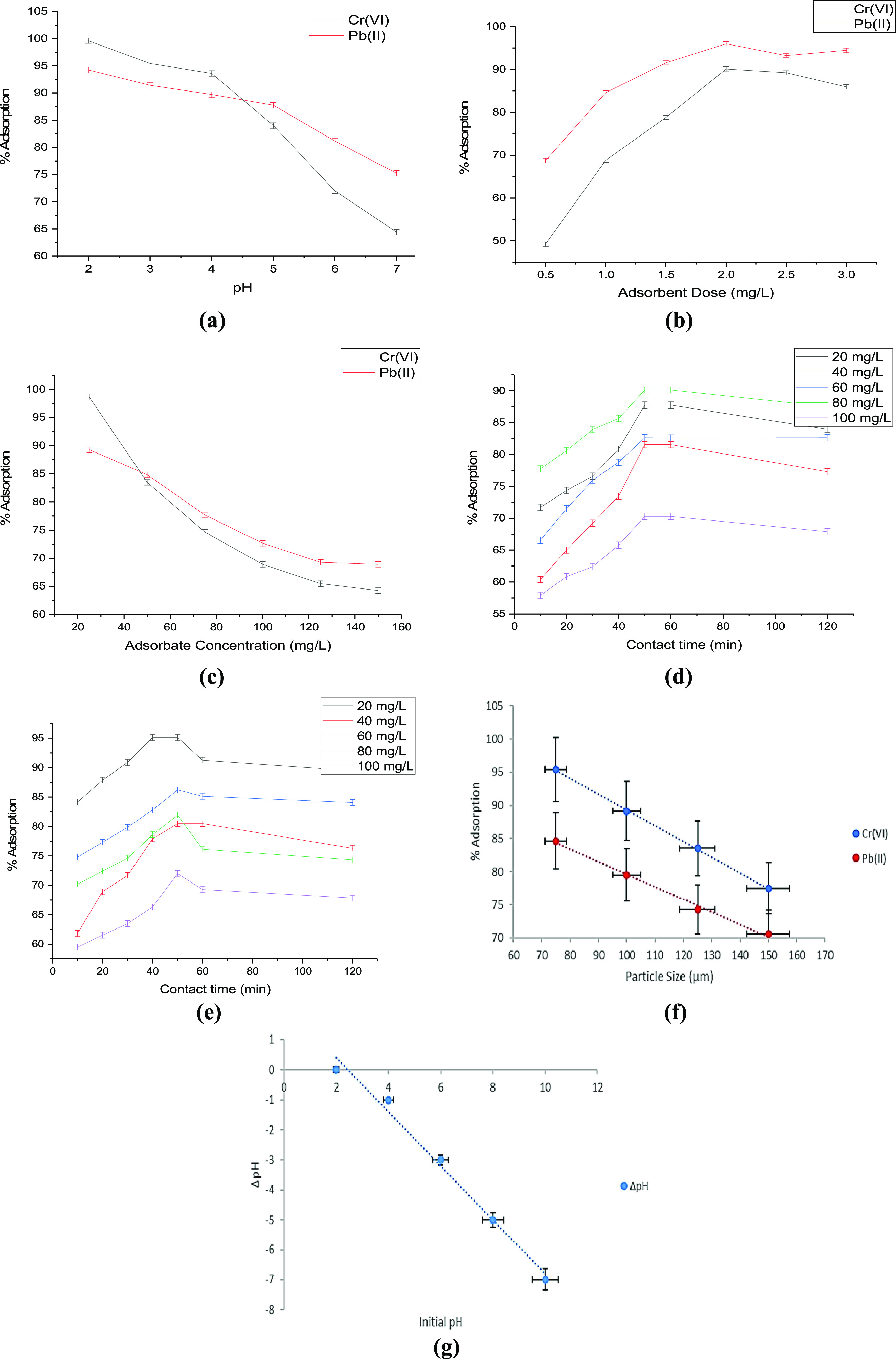
Batch mode of adsorption studies by altering (a) pH, (b) rice straw concentration, (c) ion concentration, (d) contact time for Cr(VI), (e) contact time for Pb(II), (f) particle size, and (g) pH_ZPC_.

The pH was altered by adding HNO_3_ solution, and the efficiency of targeted metal ions adsorption was examined through the batch mode of study by fixing the initial ion concentration of 20 mg/L and biosorbent dose of 2 g/L. Within 60 min of contact time, the effect of metal ion adsorption changes was examined at room temperature (25 °C) and represented in Figure 3a. The increased pH of the metal ion-containing solution increases adsorption efficiency. The adsorption process reached the maximum level at a pH of 6.0. However, any value beyond this decreased metal ion adsorption, suggesting that the adsorption process had reached its saturation point.^29^ The solution pH was more than 6.0. The highly positively charged ions were major in attracting the negatively charged metal ions in the solution. The active site availability in higher pH values was found to be low, and the rice straw adsorbent had a minimum number of active sites, which get filled up with pollutants. Moreover, at higher pH values, hydroxyl precipitation occurred, leading to a decrease in the efficiency of the adsorption process.^30^ Hence, the optimum pH for this batch adsorption study was fixed at 6.0, and 94.26% of Cr(VI) and 87.19% of Pb(II) ions were removed from the wastewater.

The metal ion adsorption was increased from 65 to 99% for Cr and from 75 to 94 for Pb, with a decrease in the solution’s pH, as shown in Figure 3a. The plot indicates the dependency of metal ions’ dependency on the aqueous solutions’ pH. The increase in pH level has decreased the efficiency of the adsorption process, and a maximum of 99.6% of Cr(VI) ions were removed from the synthetic solution at a pH of 2.0. By increasing the solution’s pH, the efficiency was reduced to around 35.22%, and higher pH ranges decreased the Cr(VI) ions because of the hexavalent state to the trivalent state. The chromium ions and their conversion in lower and moderate pH levels were described by referring to eqs 17–19. The protonation levels of the biosorbent were attributed to the increase in the amount of adsorption at lower pH levels. The neutralization happened due to the presence of negatively charged ions on the adsorbent’s surface reacting with the hydrogen ions at higher pH values. Active adsorption sites can also be developed, providing an anionic nature for Cr and Pb complexes on the surface. In addition, it was observed that the initial pH of the metal ion solution was consistently lower than the final pH of the solution after adsorption, indicating the neutralization of metal ions due to the interaction of H^+^ and negatively charged ions.^31^ Hence, the development of H^+^ ions is high with positively charged sites. A similar trend is also seen in Figure 3b for the Pb(II) metal ions.
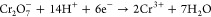
17
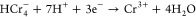
18
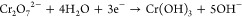
19

The higher adsorption rate of pollutants in lower pH levels can be explained based on the chemical properties of adsorbent material. If the pH level is <7.0, the chromium ions exist as Cr_2_O_7_^–^ and HCrO_4_^–^; if the pH is >7.0, they exist as HCrO_4_^–^. The ability to adsorb Cr_2_O_7_^–^ and HCrO_4_^–^ components by the rice straw adsorbent results in a very high adsorption rate. But, the adsorption rate was very low in the alkaline state due to Cr(VI) annihilation. The dichromate ion and its diffusion were reduced due to the interface of a high concentration of H^+^ ions at lower pH values. A similar trend was developed for Pb(II) metal ions also. An increased pH level decreases the level of Pb(II) metal ion adsorption due to OH^–^ competition. The increase in adsorption efficiency with decreased pH of the synthetic solution increases the hydrogen ion concentrations. The surface maintains a net positive charge with hydronium (H_3_O^+^) ions in lower pH values associated with the adsorbent surfaces.^32^ The availability of metal ions and their functional groups is the major reason for the increase in metal ion removal for adsorption. The decrease in the adsorption surface with electrical repulsion between cations with increased pH reduces the adsorbing surfaces.

### 3.3 Effect of Rice Straw Dose

The rice straw biochar material possesses numerous active sites that draw pollutants from various sources. The adsorption efficacy was determined based on the availability of these active sites. In a batch study, the impact of rice straw biochar dosage on metal ion adsorption was evaluated, ranging from 0.5 to 3.0 g/L while maintaining an optimal pH of 2.0. The analysis was performed at room temperature with an initial metal ion concentration of 50 mg/L. Figure 3b shows the impact on the adsorption efficiency by varying the rice straw biochar dose. In the initial stage, the adsorbent’s metal ion adsorption capacity increased rapidly with the adsorbent dose. The rice straw biochar dose level goes more than 2.5 g/L; there was a decrease in adsorption efficiency, which indicates the attainment of saturation level. Hence, the adsorbent’s active sites and availability reach a minimum level.^33^ Due to this, the efficiency rate decreased with an increase in the concentration of the adsorbent dose.

### 3.4 Effect of Metal Ion Concentration

At room temperature, the efficiency of metal ion adsorption was examined by altering the initial concentrations of the metal ions. Figure 3c depicts the effect of changing the metal ion concentration on adsorption efficiency. The increase in metal ion concentration decreased the adsorption efficiency and reached the saturation level at 150 mg/L concentration. At the initial concentration of 20 mg/L, the highest adsorption efficiency was observed for Cr(VI)—98.37% and Pb(II)—89.54%. As the initial concentration of metal ions increased beyond 20 mg/L, the adsorption efficiency decreased. During the higher metal ion concentrations, the active sites available in the biosorbent were extremely low, and it decreased the adsorption rate.^34^ The optimum metal ion concentration was 20 mg/L for further experimental studies. The fact that the adsorbent material’s adsorption percentage decreased as the metal ion concentration increased shows that the saturation threshold has not yet been achieved. This suggests that there are still available adsorption sites on the adsorbent’s surface that may bind and absorb more metal ions. This discovery is further supported by the consistent reduction in the adsorption quantity with rising metal ion concentration. The results also show a substantial difference in rice straw biosorbent’s adsorption properties when exposed to single-metal ions and multimetal ions.

Compared to multimetal ion systems, single-metal-ion systems have a tendency toward better adsorption efficiency. The main reason for this mismatch is that single-metal-ion adsorption scenarios exhibit higher adsorption efficiencies due to the availability of a greater number of unique adsorption sites that are specially designed to bond with the particular metal ion of interest. Adsorption efficiency is increased when there is just one metal ion in the solution because the adsorbent may devote more of its active sites to adsorbing and removing that one metal ion. However, the simultaneous presence of many metal ions in multimetal ion systems presents a competitive dynamic for the available adsorption sites, resulting in decreased adsorption effectiveness for each individual metal ion. There is increased rivalry among the metal ions to occupy the few available adsorption sites when multimetal ions are present in greater quantities.

### 3.5 Effect of Contact Time

The reaction time between the adsorbent and adsorbate is an important stage in adsorption. Taking the same concentrations of metal ions, the effect of contact time concerning adsorption was investigated at different time intervals, ranging from 10 to 120 min. The optimum pH and rice straw doses were 2.0 and 2 g/L; experimental analysis was performed at room temperature. Figure 3d,e illustrates the impact of altering the contact time and ion concentration on the adsorption efficiency for Cr(VI) and Pb(II), respectively. The increased contact time between adsorbent and adsorbate for reaction consistently increases the metal ion’s adsorption rate. The contact time reaches 50 min, and the efficiency rate starts decreasing, indicating the equilibrium stage of the adsorption process. In the earlier stages, the active site availability in the adsorbent was high, increasing the adsorption rate. As time progressed, the active sites on the adsorbent became fully saturated with pollutants, leading to decreased adsorption or metal ion uptake from the sources.^35^ The metal ion compounds were deposited on the adsorbent’s surface in the meso- and micropore area, and they became almost full during the initial period. Hence, the transfer of masses from the solid to the liquid phase was found to be extremely low, and also, these particles need to travel a longer distance with additional forces.^36^ Hence, the adsorption rate decreased after the saturation period of 50 min.

### 3.6 Effect of Particle Size Distribution

The efficiency of the adsorption process in the batch mode is influenced by variations in particle size. To assess the impact of particle size on the uptake of metal ions, the sizes of the adsorbent particles were adjusted to 75, 100, 125, and 150 μm levels. The results of this study are depicted in Figure 3f, where the effect of different particle sizes is evaluated. The remaining optimum parameters were adopted from previous studies, and their effects were analyzed across various particle sizes. According to Figure 3f, the highest adsorption rate was observed at a low particle size (75 μm), and as the particle size increased, the adsorption efficiency decreased. This phenomenon is attributed to the surface area available for adsorption. With smaller particle sizes, the surface area increases, leading to an enhanced adsorption capacity. Conversely, when the particle size increases, the surface area decreases, making it more challenging to adsorb pollutants. Consequently, the uptake of metal ions was reduced with larger particle sizes.

### 3.7 pH—Zero Point Change and Its Determination

The rice straw biosorbent showed a pHzpc value of approximately 2.133 (Figure 3g), which was determined using the procedure described in Section 2.7. This pHzpc value provides valuable insight into the adsorption behavior of the adsorbent under different pH conditions. Under basic pH conditions, the adsorbent’s surface charge becomes negative due to the pH being above the pHzpc value. As a result, cations such as Cr(VI) and Pb(II) are favorably adsorbed onto the adsorbent. On the other hand, at pH values below the pH_zpc_, the surface charge of the adsorbent becomes positive, leading to the adsorption of anions. The adsorption conditions were found to be most favorable for Cr(VI) and Pb(II) ions under basic pH conditions, which is consistent with the earlier discussion. The pH_zpc_ value helps explain why the rice straw biosorbent exhibited better performance under basic pH conditions for the adsorption of these specific pollutant ions.

### 3.8 Adsorption Isotherm studies

#### 3.8.1 Langmuir Study

**Figure 4:**
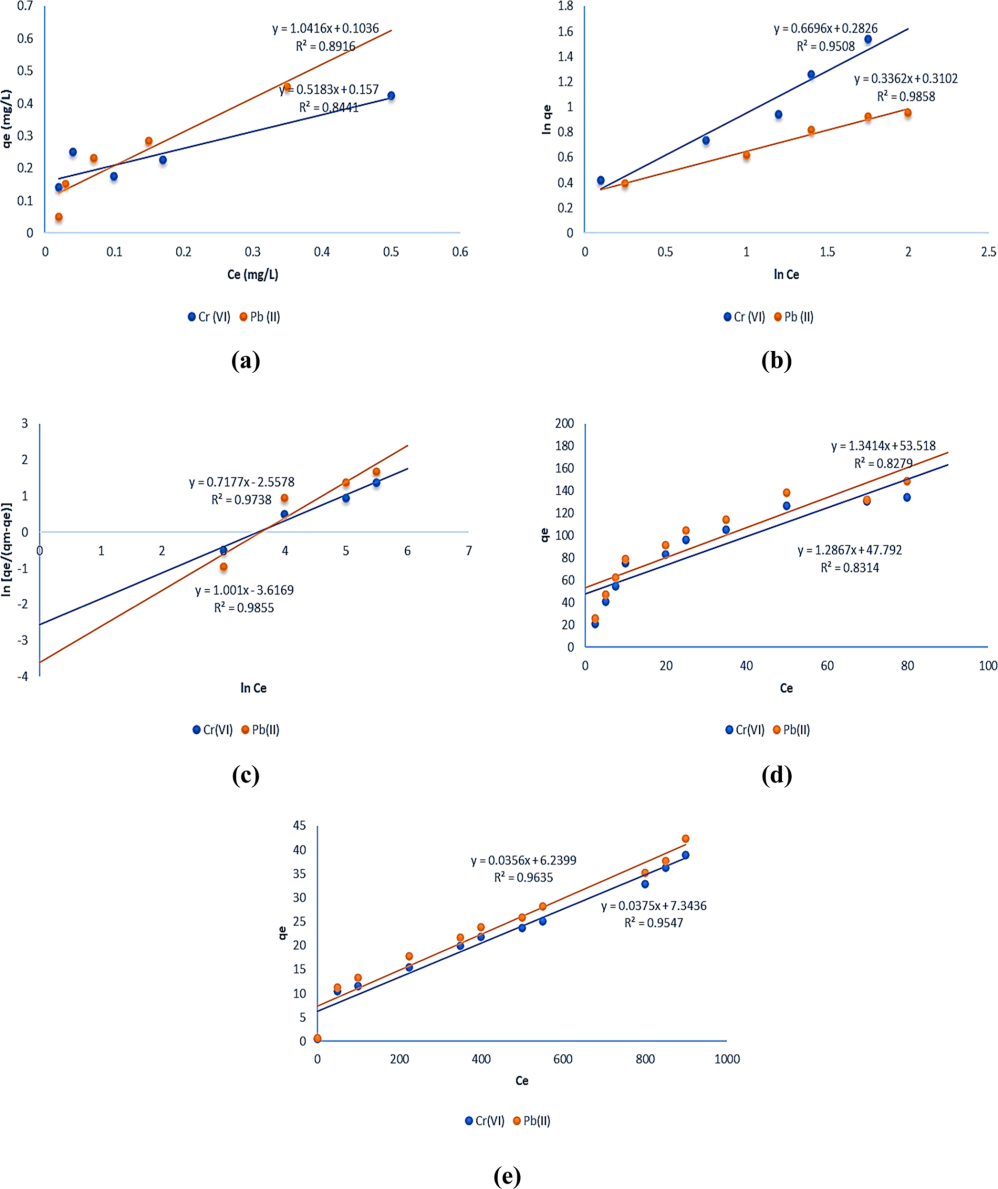
(a) Langmuir, (b) Freundlich, (c) Sips, (d) Toth, and (e) Fritz–Schlunder—isothermal linear plots for chromium and lead adsorption.

Langmuir isotherm studies were conducted to investigate the transfer of metal ions by the adsorbent concerning solid and liquid. Langmuir isotherm studies were used to identify the monolayer adsorption process by physical forces.^37^ The uniform adsorption of metal ions was determined by creating a linear plot of *C*_e_/*q*_e_ against *C*_e_. Figure 4a shows the linear plots obtained using the Langmuir isotherm model to analyze chromium adsorption and lead heavy-metal ions. The regression values (*R*^2^) for each plot are also displayed. However, the *R*^2^ values for each plot were less than 0.95, indicating that the Langmuir isotherm model was not applicable. The isotherm model constants were evaluated and are presented in Table 2, but these values did not align with the adsorption process. To evaluate the metal ion adsorption process, the separation parameter value, which ranges from 0 to 1 and indicates the effectiveness of the adsorption process by the adsorbent, was utilized. Based on this experimental study, it was determined that the adsorption of metal ions by the rice straw adsorbent did not follow a monolayer process with a physical mode of heterogeneous activity.

**Table 2:** Adsorption Isothermal Constants for Metal Ion Adsorption

			calculated values	
s. no.	model	parameters	Cr(VI)	Pb(II)
1.	Langmuir	*q*_max_	11.293	9.348
		*K*_L_	0.419	0.501
		*R*^2^	0.891	0.844
2.	Freundlich	*K*_f_	3.192	2.947
		*n*	3.029	2.847
		*R*^2^	0.950	0.985
3.	Sips	*K*_S_	16.823	8.481
		β_S_	1.492	1.753
		*a*_S_	0.601	0.348
		*R*^2^	0.973	0.985
4.	Toth	*Q*_max_	31.273	28.481
		*b*_T_	0.453	0.249
		*n*_T_	0.973	0.789
		*R*^2^	0.831	0.827
5.	Fritz–Schlunder	*Q*_MFS_	0.438	0.298
		*K*_FS_	57.373	194.823
		*N*_FS_	1.127	0.654
		*R*^2^	0.954	0.963

#### 3.8.2 Freundlich Study

Freundlich isotherm studies have evaluated the multilayer adsorption process by chemical mode by plotting the linear plots of ln *C*_e_ vs ln *q*_*e*_. Figure 4b depicts the linear plots of chromium and lead heavy-metal ions obtained from Freundlich isotherm studies and the corresponding regression values. The high *R*^2^ values (>0.95) suggest the appropriateness of Freundlich isotherm studies for analyzing the adsorption of these metal ions. The values of the constants have been evaluated and are listed in Table 2. The expected values nearly correlated with the calculated values, and the calculated values fitted well with the metal ion adsorption. The initial analysis confirmed that the adsorption of metal ions by the rice straw adsorbent followed a chemical mode and dynamic multilayer adsorption.^38^ Also, the heterogeneous nature of adsorption happened during the time of pollutant uptake by the biosorbent.

#### 3.8.3 Sips Isotherm

Figure 4c represents the Sips model isotherm plots, which show the applicability of the isothermal model in the biosorption process. By analyzing the heterogeneity factor (*n*) value from the linear plots of the model, it was possible to determine whether the adsorption process was homogeneous or heterogeneous.^39^ The plots of Sips model studies and their regression (*R*^2^) values were found to be high, indicating this model’s suitability. The “*n*” value, ranging from 0 to 1, was used to determine whether the Langmuir or Freundlich isotherm fit was appropriate. If *n* > 1, the process fitted with Freundlich isotherm fit.

#### 3.8.4 Toth Isotherm

Based on its constant values, the Toth isotherm model was used to identify the heterogeneous solid surface. The constants (*Q*_max_, *b*_T_, and *n*_T_) were obtained and are listed in Table 3 with the help of isothermal plots shown in Figure 4d. This three-parameter model is known for providing a high accuracy of isotherm fitting. The interaction between the adsorbent surfaces and heavy-metal pollutants was analyzed using this model, but the plots provided very low regression (*R*^2^ < 0.95) concerning the biosorption process and did not fit well with the adsorption process. The Toth isotherm model is used to fit the equilibrium data in cases where the Langmuir isotherm model is not applicable to describe the adsorption process.^40^ However, in this study, the Langmuir data fit well with the equilibrium data, indicating that the Toth isotherm study is not necessary to check the favorable fitting of the adsorption process.

**Table 3:** Constants of Adsorption Kinetics for Metal Ion Removal Using a Rice Straw Biosorbent

			pseudo-first-order				pseudo-second-order				Elovich			Boyd			intraparticle diffusion	
s. no.	type of metal	conc. (mg/L)	*K* (min^–1^)	*q*_e_, cal (mg/g)	*R*^2^	*K* (g/mg· min) × 10^–3^	*q*_e_, cal (mg/g)	*h* (mg/g·min)	R^2^	*a* (mg/g· min)	*b* (g/mg)	*R*^2^	*B*	*D*_i_ (× 10^–3^ m^2^/s)	*R*^2^	*K*_p_ (mg/g·min^1/2^)	*C*	*R*^2^
1.	Cr(VI)	25	0.034	2.64	0.95	16.69	2.15	0.10	0.96	0.244	1.55	0.94	0.034	5.472	0.915	0.197	0.41	0.95
2.		50	0.043	7.02	0.93	5.73	5.19	0.18	0.98	0.988	0.74	0.93	0.044	7.340	0.973	0.399	0.58	0.95
3.		75	0.041	10.00	0.93	3.34	8.30	0.22	0.98	0.978	0.55	0.92	0.043	7.621	0.963	0.517	0.60	0.98
4.		100	0.039	11.36	0.94	5.07	10.75	0.26	0.98	0.948	0.44	0.95	0.039	6.856	0.914	0.783	0.54	0.99
5.		125	0.048	17.47	0.92	2.00	12.91	0.29	0.97	0.977	0.35	0.92	0.049	8.725	0.982	0.804	0.37	0.93
6.		150	0.045	19.43	0.93	3.12	13.82	0.30	0.96	0.934	0.26	0.92	0.045	7.452	0.952	0.856	0.32	0.96
7.	Pb(II)	25	0.046	3.68	0.91	12.62	2.70	0.10	0.97	0.263	1.16	0.91	0.046	7.678	0.943	0.190	0.38	0.98
8.		50	0.041	6.54	0.93	5.32	5.47	0.12	0.98	0.328	0.81	0.94	0.041	6.294	0.983	0.348	0.51	0.94
9.		75	0.043	9.95	0.92	3.56	8.60	0.23	0.98	0.541	0.65	0.93	0.045	7.959	0.924	0.554	0.68	0.95
10.		100	0.046	12.38	0.94	2.77	10.10	0.28	0.97	0.611	0.47	0.92	0.046	7.678	0.983	0.686	0.67	0.95
11.		125	0.052	20.55	0.92	2.30	11.56	0.31	0.98	0.618	0.31	0.90	0.053	8.590	0.941	0.772	0.51	0.97
12.		150	0.050	25.48	0.94	2.65	12.73	0.33	0.96	0.596	0.20	0.92	0.055	8.972	0.922	0.805	0.32	0.96

#### 3.8.5 Fritz–Schlunder Isotherm

The adsorption process was analyzed with respect to temperature and pressure variations by using the Fritz–Schlunder four-parameter isotherm model. The model’s linear plots are presented in Figure 4e, and the obtained constants are listed in Table 2. The Fritz–Schlunder isothermal model fitted with the biosorption process by referring to the higher regression values (*R*^2^ > 0.95) obtained from each plot in Figure 4e. The constants obtained from the plots agree well with the adsorption process. These isotherm studies were used to determine whether the adsorption process was favorable or not. Based on the studies, it was found that the Langmuir, Freundlich, R-P, Sips, and Fritz–Schlunder models fit well with the azo dye adsorption process, confirming the monolayer adsorption in a heterogeneous nature.^41^

### 3.9 Kinetic Studies

The initial metal ion concentrations in the wastewater were adjusted, and the kinetic studies were performed at different time intervals. Pseudo-first and second-order studies were performed to determine the nature of adsorption, whether physical or chemical. These studies are crucial in determining whether strong or weak forces govern Cr(VI) and Pb(II) adsorption using the rice straw biochar material.

#### 3.9.1 Pseudo-First-Order (PFO) studies

**Figure 5:**
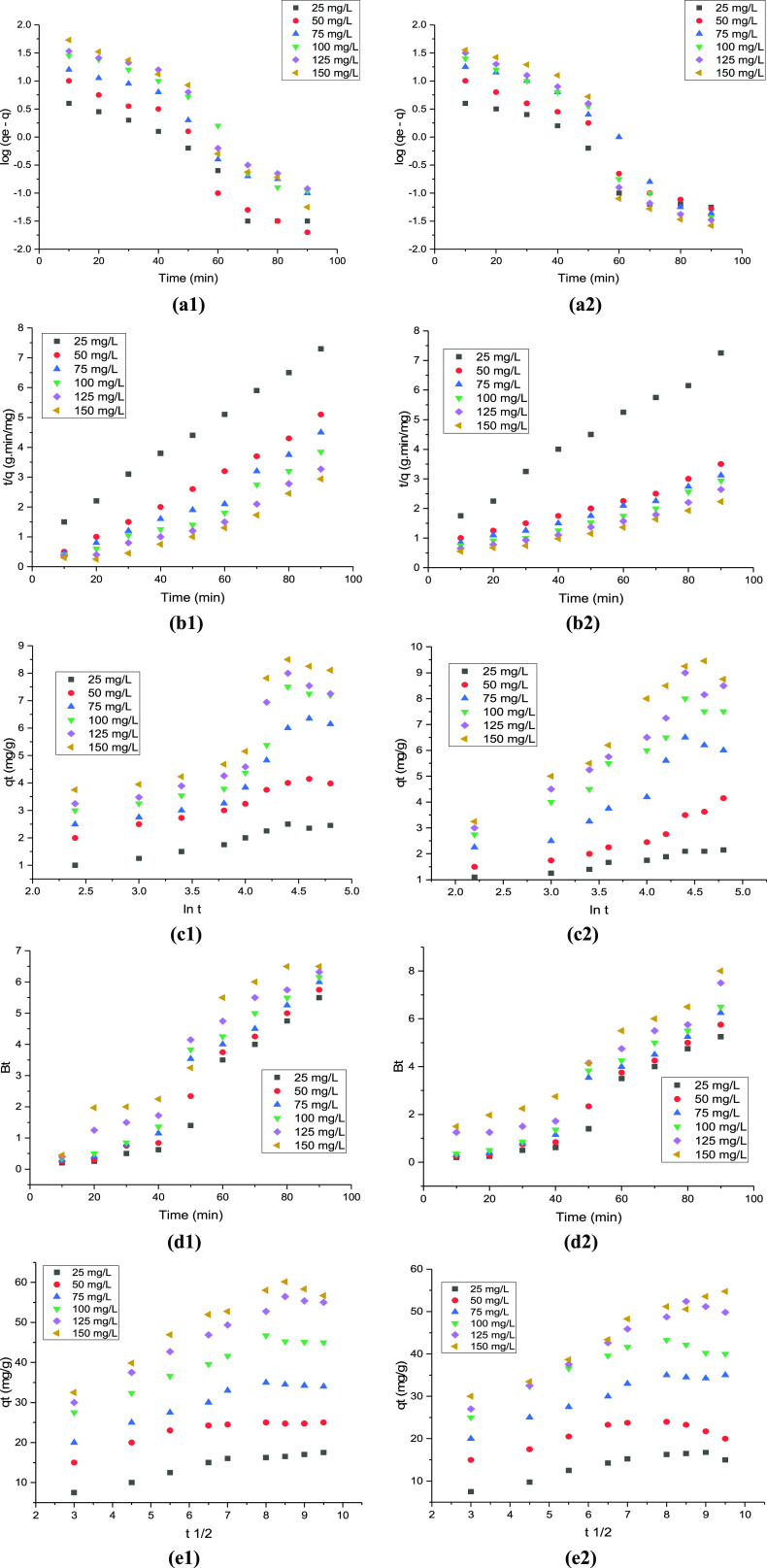
(a1, a2) Pseudo-first-order, (b1, b2) pseudo-second-order, (c1, c2) Elovich, (d1, d2) Boyd, and (e1, e2) IPD kinetic plots for (a) Cr(VI) and (b) Pb(II) metal ion adsorption.

Referring to eq 8, the linear plots were obtained for the PFO reaction and are shown in Figure 5a1,a2 for Cr(VI) and Pb(II) ions, which is used to analyze the mechanism of the metal ion adsorption by the adsorbent material. The regression values for each plot were obtained from the linear fit and are presented in Table 3, along with the kinetic constants. However, the obtained values of these kinetic constants did not align well with the physical mode of the adsorption process, as indicated by the low regression values for both metal ions (*R*^2^ < 0.95). The attainment of the saturation point indicates that the metal ion uptake process has reached a steady state, and the values obtained from each linear plot suggest that the PFO studies did not fit well with the adsorption process. Hence, the process of metal ion adsorption was not followed by physical mode.^42^

#### 3.9.2 Pseudo-Second-Order (PSO) Studies

To explore the chemical mode of the adsorption process, a range of PSO model studies were carried out by altering the ion concentrations in the aqueous solutions, which ranged from 20 to 100 mg/L. PSO plots for Cr and Pb metal ions are shown in Figure 5b1,b2, respectively. The respective constant values for second-order kinetics were calculated and are presented in Table 3. It is worth mentioning that the regression value for each linear plot of chromium and lead metal ions was found to be high, and the constants derived from these plots were found to be in line with the adsorption process.^43^ The suitability of the pseudo-second-order kinetic model is confirmed by the *R*^2^ value exceeding 0.95, indicating the chemical adsorption process by the biosorbent.

#### 3.9.3 Elovich Kinetic Study

The adsorption kinetics of pollutant uptake by the biosorbent were evaluated through Elovich kinetic model studies. Figure 5c1,c2 depicts the Elovich model kinetic plots, which were used to examine the adsorption behavior of rice straw biosorbent. The constants a and b represented in Table 3 indicate the nonapplicability of this kinetic model. Although very low regression values were obtained from each plot, they were found to be low (*R*^2^ < 0.95), and the constant values are not in agreement with the pseudo-second-order studies. Elovich’s model can be used to explain the heterogeneous adsorbents during adsorption.^44^

#### 3.9.4 Boyd Kinetic Study

The linearity and experimental variables were checked using *B*_t_ versus *t* plots. In the case where the plots are linear and pass through the origin, it indicates that intraparticle diffusion is the rate-limiting step in the adsorption process. Conversely, if the plots deviate from linearity or do not pass through the origin, intraparticle diffusion is not the slowest stage. Figure 5d1,d2 shows the Boyd model kinetic plots in various concentrations of metal ions, and these plots do not move into the origin, which confirms the regulation of external or film diffusion of the adsorption process by rice straw biosorbent.^45^ The low regression coefficient values for each plot indicate that the Boyd kinetic model cannot be applied to the metal ion adsorption process of the rice straw biosorbent.

#### 3.9.5 IPD Kinetic Study

The IPD kinetic studies were investigated by altering the metal ion concentrations, and the graphical representation of these kinetic studies is shown in Figure 5e1,e2, respectively. The obtained kinetic constants of this study are presented in Table 3, derived from the corresponding plots. If the plot of metal ion uptake versus time by rice straw biosorbent passes through the origin point, it indicates that the process of metal ion adsorption is controlled by intraparticle diffusion.^46^ The adsorption process is affected by two or more phases if the multilinear curve pattern is developed. However, the plots of IPD studies (Figure 5e1,e2) show the curve in dual structure with different degrees for various concentrations of Cr and Pb metal ions during the starting and final stages. The metal ion adsorption process can be attributed to the following assumption based on the observed phenomenon: Initially, the adsorption is primarily influenced by the boundary layer effect, and subsequently, it is governed by intraparticle diffusion.

### 3.10 Thermodynamic Studies

**Figure 6:**
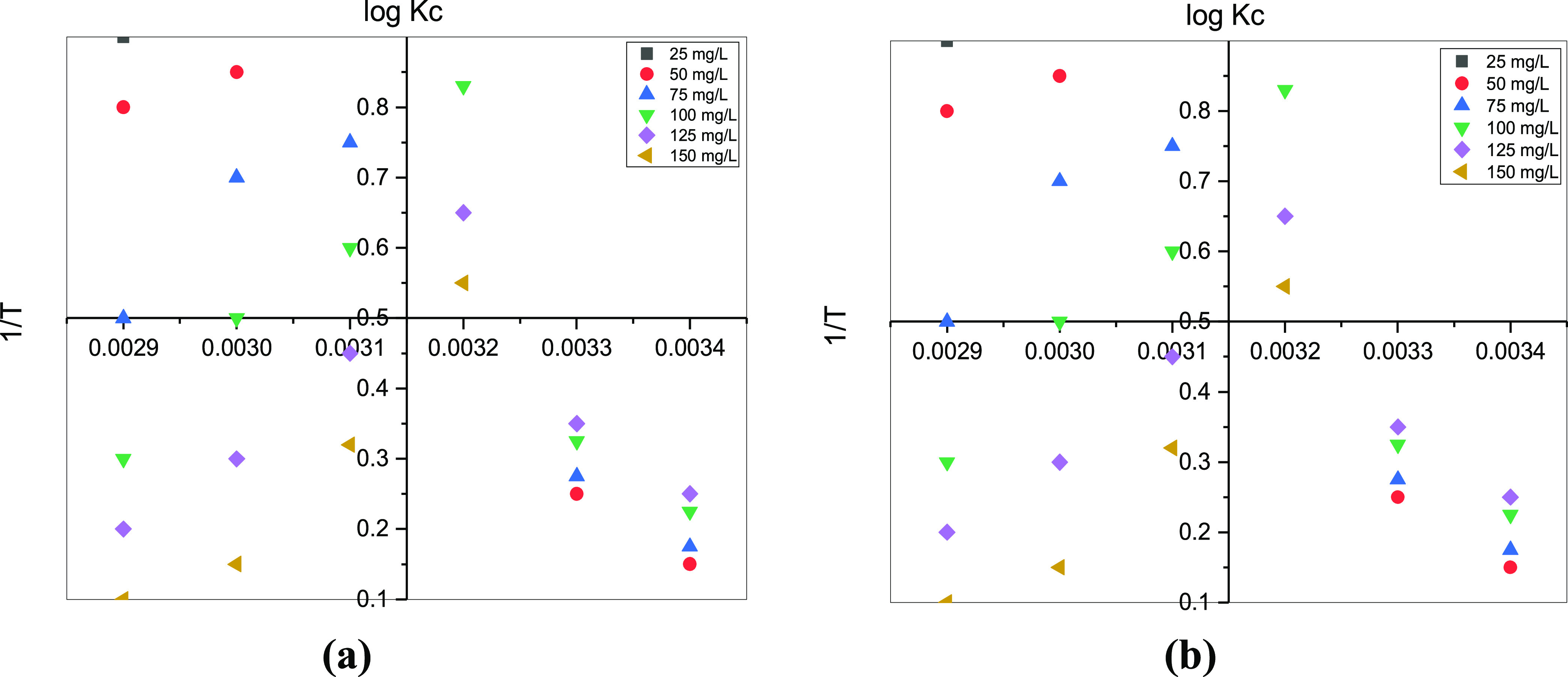
Thermodynamics of (a) Cr and (b) Pb uptake using a rice straw biosorbent.

Thermodynamic studies have determined the spontaneous nature of the metal ion adsorption process under controlled temperatures by examining whether the process is exothermic or endothermic. This study evaluated the thermodynamic nature of the adsorption process at different temperatures and different metal ion concentrations. Figure 6a,b shows the thermodynamic plots for Cr(VI) and Pb(II) metal ions with concentrations of 25, 50, 75, 100, 125, and 150 mg/L, respectively. The value of enthalpy and entropy (Δ*H*_0_ and Δ*S*_0_) was calculated from each plot in Figure 6 and represented in Table 4. The Gibbs energy (Δ*G*_0_) of the metal ion adsorption process was also obtained from the plot to identify the rice straw biochar material and its nature. The negative Gibbs energy values observed in Table 4 indicate that the process of metal ion adsorption is spontaneous, and the endothermic nature of the process can also be inferred from the positive Δ*H*_0_ values observed. Also, the rice straw biochar adsorbent has a solid and liquid uncertainty nature, which was identified by positive entropy values.^47^ The above studies confirm that the metal ion adsorption process follows the endothermic spontaneous nature of the adsorbent material.

**Table 4:** Thermodynamic Constants for Metal Ion Adsorption Using a Rice Straw Adsorbent

			Gibbs energy (Δ*G*_0_) kJ/mol			
metal ion concentration, mg/L	enthalpy (Δ*H*°) kJ/mol	entropy (Δ*S*°) J/mol·K	20 °C	30 °C	40 °C	50 °C
Cr(VI)						
25	83.482	189.320	–14.293	–10.042	–9.025	–7.203
50	41.294	99.042	–10.452	–8.204	–7.239	–5.928
75	25.493	49.023	–8.296	–7.204	–6.245	–5.293
100	16.395	33.842	–5.395	–5.121	–4.921	–4.548
125	12.041	29.456	–3.534	–3.252	–3.576	–3.201
150	8.945	25.923	–2.042	–1.943	–1.843	–1.652
Pb(II)						
25	73.945	163.494	–12.943	–10.943	–8.239	–7.543
50	39.298	83.723	–10.284	–9.204	–7.283	–5.892
75	24.035	40.294	–8.593	–8.023	–6.032	–4.925
100	18.204	21.049	–6.402	–5.394	–4.785	–3.024
125	13.543	16.305	–4.203	–3.253	–3.012	–2.435
150	9.836	10.423	–3.021	–2.893	–2.324	–1.936

## 4 Adsorption Mechanism

**Figure 7:**
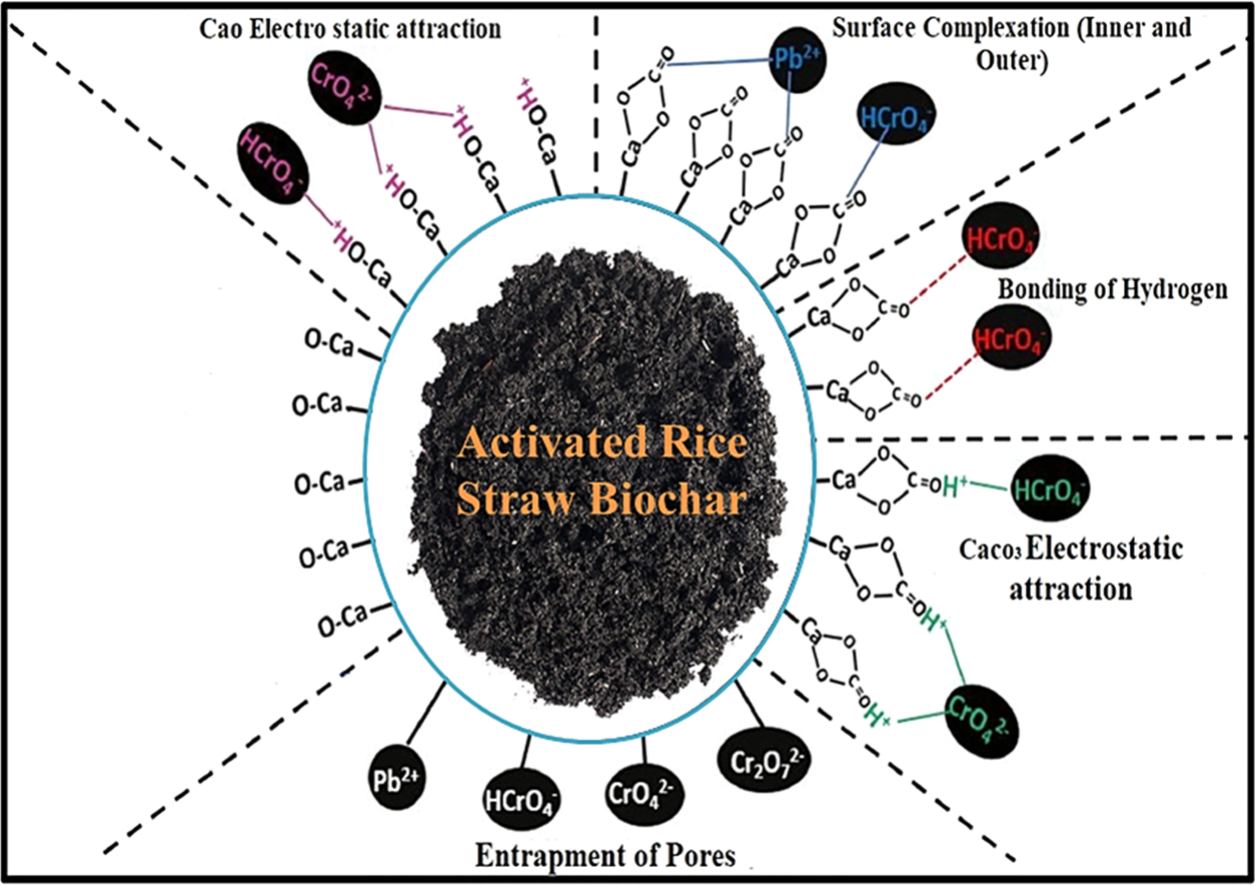
Adsorption mechanism of the targeted metal ions.

Figure 7 illustrates the mechanism of metal ion adsorption by the rice straw biochar adsorbent examined in this study. The metal ion uptake process by the adsorbent follows intraparticle diffusion/external film diffusion. Under extraordinary conditions, external film and intraparticle diffusion may happen simultaneously during adsorption. In this experimental study, three different types of stages in the adsorption process were observed. In the first stage, external film diffusion was observed, and the Cr(VI) and Pb(II) metal ion adsorption and its movement within the rice straw adsorbent was identified as a result of external forces. In stage two, the particle diffusion moved the pollutants into the inner sides of the adsorbate material. In stage three, the deep penetration of metal ions into the adsorbate happened due to the availability of binding spaces and penetration speed.

The adsorption mechanism for Cr(VI) and Pb(II) ions on the adsorbents involved synergistic processes, as shown in Figure 7. The surface complexation was attributed to the carbonate (–C=O) functional groups toward metal ions and its adsorption; the π bond of the –C=O group formed a bond with the targeted heavy-metal ions. The FTIR results also show evidence of bonding (Figure 2a1,a2) where the peak for −CO_3_^2–^ groups decreased after the process of metal ion adsorption, representing the −CO_3_^2–^ involvement in metal ion uptake.^48^ Hydrogen bonding also developed due to the interaction between the –C=O oxygen and HC_r_O_4_^–^ hydrogen. The adsorbent surface became protonated and positively charged in acidic conditions, leading to the electrostatic attraction between the adsorbent and Cr(VI). The mechanism of metal ion adsorption was intraparticle diffusion, which involved the Cr(VI) and Pb(II) ion entrapment. When the pH goes to a strong alkaline stage, a precipitate of CaCr_2_O_7_ was formed in yellow on the surface of the adsorbent.

## 5 Reusability and Regeneration Studies

**Figure 8:**
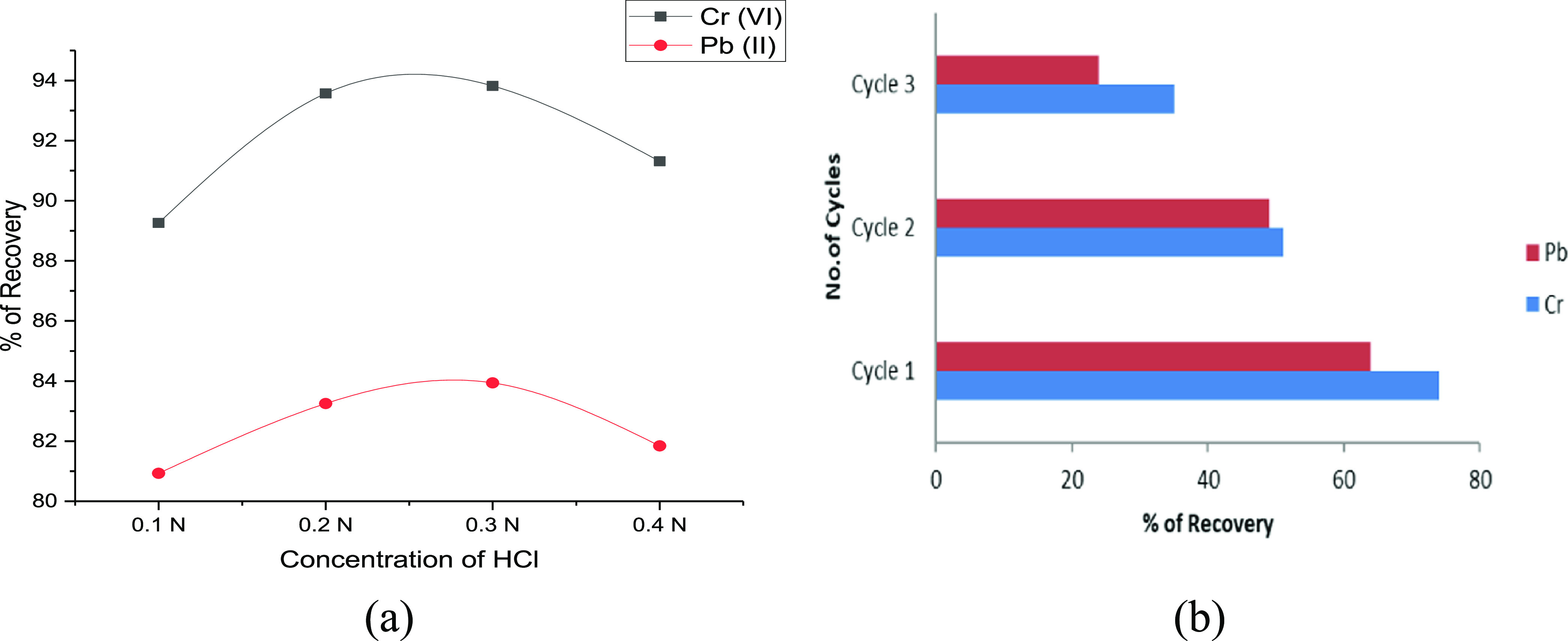
(a) Desorption and (b) recycling studies of metal ion adsorption.

The rate of desorption of metal ions by adding HCl is shown in Figure 8a. At the beginning of the desorption process, the recovery of metal ions was rapid and increased with the concentration of hydrochloric acid. The desorption rate eventually reached saturation upon the addition of 0.3 N hydrochloric acid to the solution. Further increases in the hydrochloric acid concentration reduce the desorption rate, indicating the equilibrium level of attainment. The exhausted rice straw biosorbent and releasing capacity are directly proportional to the desorption rate. Due to this reason, the desorption rate decreased with an increase in concentration.^49^ To check the performance of the adsorbent, multiple cycles of analysis were conducted. Referring to Figure 8b, during the first cycle of regeneration, the recovery of metal ions was high, and the increase in the number of cycles decreased the recovery rate. Around 74.17% of Cr(VI) and 63.29% of Pb(II) were recovered from this study. Table 5 represents the comparison studies to validate the obtained results from the batch studies.

**Table 5:** Comparison of Experimental Results with Other Research Works Using Rice Husk and Straw Material as a Biosorbent

metal	eff. (%)	optimum pH	optimum Dose	initial metal ion concentration	contact time	isotherm fit	kinetic fit	refs
Cr and Pb	95.57 and 85.68	6.0	2.5 g/L	25 mg/L	50 min	Freundlich	pseudo-second-order	in this study
Cd and Zn	20.9 and 8.4	5.0 and 4.0	0.1 and 0.55 g	15 mg/L	30 min	Freundlich	pseudo-second-order	(50)
Pb and Cu	96.25 and 75.54	-	0.13 g	100 mg/L and 60 mg/L	41.96 s and 59.35 s	Langmuir	pseudo-second-order	(51)
Cd	71.4	7.0	0.6 mg/L	800 mg/L	240 min	Langmuir, Freundlich, and Temkin	-	(26)
Pb	42.55	5.5	4 g/L	40 mg/L	30 min	Langmuir and Freundlich	-	(52)
Cu	88.9	4.0	2 g/L	5 mg/L	180 min	Langmuir and Freundlich	pseudo-second-order	(53)
Cr, Pb and Zn	87.12, 88.63, 99.28	6.0	2.5 g/L	25 mg/L	60 min	Temkin and D-R	Elovich	(54)

## 6 Economic and Application Feasibility of Developed Biochar

In India, rice straw biochars present a cost-effective solution for water treatment because of their abundant availability as a byproduct of rice cultivation. These biochars exhibit a high adsorption capacity and effectively remove a wide range of contaminants from water. The porous structure of biochars, coupled with their large surface area, enables efficient adsorption processes. Moreover, their potential for regeneration and reuse further enhances their cost-effectiveness, making them an attractive option for water treatment in India. In the present study, desorption studies showed that 92.17% of Cr(VI) and 82.34% of Pb(II) could be recovered by using concentrated hydrochloric acid, allowing the biochar to be reused for up to three cycles. In subsequent cycles, the biochar demonstrated a removal efficiency of less than 63% for both metal ions. Overall, the availability of rice straw as a raw material, combined with the efficient adsorption capabilities and potential for regeneration, makes rice straw biochars a cost-effective option for water treatment applications in India. Their utilization presents a sustainable and economically viable solution for addressing water pollution challenges in the country.

## 7 Conclusions

Batch adsorption studies were conducted to evaluate the effectiveness of the rice straw biosorbent in removing chromium and lead metal ions from wastewater. The experiments were carried out at room temperature (25 °C). The Freundlich, Sips, and Toth isothermal studies indicated a multilayer adsorption process with a heterogeneous nature. The PSO, Boyd, and IPD kinetic studies confirmed that the uptake of metal ions by the rice straw adsorbent followed a chemical adsorption process. Thermodynamic studies revealed the endothermic nature of the metal ion adsorption process. Desorption studies demonstrated that concentrated hydrochloric acid could recover 92.17% of C(VI) and 82.34% of Pb(II). The biochar could be reused for up to three cycles without a significant loss of its removal efficiency (less than 63%) for both metal ions. The availability of rice straw as a raw material, along with its efficient adsorption capabilities and potential for regeneration, makes rice straw biochars a cost-effective solution for water treatment applications in India. Utilizing rice straw biochar presents a sustainable and economically viable approach to addressing water pollution challenges.

## References

[ref1] Ambaye T. G.; Vaccari M.; van Hullebusch E. D.; Amrane A.; Rtimi S. Mechanisms and Adsorption Capacities of Biochar for the Removal of Organic and Inorganic Pollutants from Industrial Wastewater. Int. J. Environ. Sci. Technol. 2021, 18 (10), 3273–3294. 10.1007/s13762-020-03060-w.

[ref2] Eleryan A.; Aigbe U. O.; Ukhurebor K. E.; Onyancha R. B.; Eldeeb T. M.; El-Nemr M. A.; Hassaan M. A.; Ragab S.; Osibote O. A.; Kusuma H. S.; Darmokoesoemo H.; El Nemr A. Copper(II) Ion Removal by Chemically and Physically Modified Sawdust Biochar; Springer Berlin Heidelberg, 2022. 10.1007/s13399-022-02918-y.

[ref3] Fideles R. A.; Teodoro F. S.; Xavier A. L. P.; Adarme O. F. H.; Gil L. F.; Gurgel L. V. A. Trimellitated Sugarcane Bagasse: A Versatile Adsorbent for Removal of Cationic Dyes from Aqueous Solution. Part II: Batch and Continuous Adsorption in a Bicomponent System. J. Colloid Interface Sci. 2019, 552, 752–763. 10.1016/j.jcis.2019.05.089. 31176922

[ref4] Venkatraman Y.; Priya A. K. Removal of Heavy Metal Ion Concentrations from the Wastewater Using Tobacco Leaves Coated with Iron Oxide Nanoparticles. Int. J. Environ. Sci. Technol. 2022, 19 (4), 2721–2736. 10.1007/s13762-021-03202-8.

[ref5] Marciniak M.; Goscianska J.; Norman M.; Jesionowski T.; Bazan-Wozniak A.; Pietrzak R. Equilibrium, Kinetic, and Thermodynamic Studies on Adsorption of Rhodamine B from Aqueous Solutions Using Oxidized Mesoporous Carbons. Materials. 2022, 15 ( (16), ). 5573 10.3390/ma15165573. 36013711 PMC9412670

[ref6] Revellame E. D.; Fortela D. L.; Sharp W.; Hernandez R.; Zappi M. E. Adsorption Kinetic Modeling Using Pseudo-First Order and Pseudo-Second Order Rate Laws: A Review. Cleaner Eng. Technol. 2020, 1 (October), 100032 10.1016/j.clet.2020.100032.

[ref7] Yogeshwaran V.; Priya A. K. Experimental Studies on the Removal of Heavy Metal Ion Concentration Using Sugarcane Bagasse in Batch Adsorption Process. Desalin. Water Treat. 2021, 224, 256–272. 10.5004/dwt.2021.27160.

[ref8] Yaseen D. A.; Scholz M. Textile Dye Wastewater Characteristics and Constituents of Synthetic Effluents: A Critical Review; Springer Berlin Heidelberg, 2019; Vol. 16. 10.1007/s13762-018-2130-z.

[ref9] Yu H.; Wang J.; Yu J.-x.; Wang Y.; Chi R.-a. Adsorption Performance and Stability of the Modified Straws and Their Extracts of Cellulose, Lignin, and Hemicellulose for Pb2+: PH Effect. Arab. J. Chem. 2020, 13 (12), 9019–9033. 10.1016/j.arabjc.2020.10.024.

[ref10] Bhadoria P.; Shrivastava M.; Khandelwal A.; Das R.; Langyan S.; Rohatgi B.; Singh R. Preparation of Modified Rice Straw-Based Bio-Adsorbents for the Improved Removal of Heavy Metals from Wastewater. Sustainable Chem. Pharm. 2022, 29 (June), 100742 10.1016/j.scp.2022.100742.

[ref11] Lee S. M.; Lee S. H.; Roh J. S. Analysis of Activation Process of Carbon Black Based on Structural Parameters Obtained by XRD Analysis. Crystals 2021, 11 (2), 153 10.3390/cryst11020153.

[ref12] Al-Ghouti M. A.; Da’ana D. A. Guidelines for the Use and Interpretation of Adsorption Isotherm Models: A Review. J. Hazard. Mater. 2020, 393 (February), 122383 10.1016/j.jhazmat.2020.122383. 32369889

[ref13] Sipos P. Searching for Optimum Adsorption Curve for Metal Sorption on Soils: Comparison of Various Isotherm Models Fitted by Different Error Functions. SN Appl. Sci. 2021, 3 (3), 387 10.1007/s42452-021-04383-0.

[ref14] Jan S. U.; Ahmad A.; Khan A. A.; Melhi S.; Ahmad I.; Sun G.; Chen C. M.; Ahmad R. Removal of Azo Dye from Aqueous Solution by a Low-Cost Activated Carbon Prepared from Coal: Adsorption Kinetics, Isotherms Study, and DFT Simulation. Environ. Sci. Pollut. Res. 2021, 28 (8), 10234–10247. 10.1007/s11356-020-11344-4. 33170468

[ref15] Khan T. A.; Nouman M.; Dua D.; Khan S. A.; Alharthi S. S. Adsorptive Scavenging of Cationic Dyes from Aquatic Phase by H3PO4 Activated Indian Jujube (Ziziphus Mauritiana) Seeds Based Activated Carbon: Isotherm, Kinetics, and Thermodynamic Study. J. Saudi Chem. Soc. 2022, 26 (2), 101417 10.1016/j.jscs.2021.101417.

[ref16] Sheeja J.; Sampath K.; Kesavasamy R. Experimental Investigations on Adsorption of Reactive Toxic Dyes Using Hedyotis Umbellate Activated Carbon. Adsorpt. Sci. Technol. 2021, 2021. 1 10.1155/2021/5035539.

[ref17] Manjuladevi M.; Anitha R.; Manonmani S. Kinetic Study on Adsorption of Cr(VI), Ni(II), Cd(II) and Pb(II) Ions from Aqueous Solutions Using Activated Carbon Prepared from Cucumis Melo Peel. Appl. Water Sci. 2018, 8 (1), 36 10.1007/s13201-018-0674-1.

[ref18] Khamwichit A.; Dechapanya W.; Dechapanya W. Adsorption Kinetics and Isotherms of Binary Metal Ion Aqueous Solution Using Untreated Venus Shell. Heliyon 2022, 8 (6), e09610 10.1016/j.heliyon.2022.e09610. 35706950 PMC9189894

[ref19] Su L.; Zhang H.; Oh K.; Liu N.; Luo Y.; Cheng H.; Zhang G.; He X. Activated Biochar Derived from Spent Auricularia Auricula Substrate for the Efficient Adsorption of Cationic Azo Dyes from Single and Binary Adsorptive Systems. Water Sci. Technol. 2021, 84 (1), 101–121. 10.2166/wst.2021.222. 34280158

[ref20] Kumar A.; Jena H. M. Preparation and Characterization of High Surface Area Activated Carbon from Fox Nut (Euryale Ferox) Shell by Chemical Activation with H3PO4. Results Phys. 2016, 6, 651–658. 10.1016/j.rinp.2016.09.012.

[ref21] Hasdemir İ. M.; Yılmazoğlu E.; Güngör S.; Hasdemir B. Adsorption of Acetic Acid onto Activated Carbons Produced from Hazelnut Shell, Orange Peel, and Melon Seeds. Appl. Water Sci. 2022, 12 (12), 271 10.1007/s13201-022-01797-y.

[ref22] Bergna D.; Varila T.; Romar H.; Lassi U. Comparison of the Properties of Activated Carbons Produced in One-Stage and Two-Stage Processes. C 2018, 4 (3), 41 10.3390/c4030041.

[ref23] Ramya D.; Thatheyus A. J.; Juliana S. J. B.; Kiruba N. J. M.; Selvam A D. G. Physical Characterization and Kinetic Studies of Zn (II) Biosorption by Morganella Morganii ACZ05. Water Sci. Technol. 2022, 85 (4), 970–986. 10.2166/wst.2022.031. 35228348

[ref24] Kurniawan A.; Ulfa S. M.; Chamidah C. The Biosorption of Copper(II) Using a Natural Biofilm Formed on the Stones from the Metro River, Malang City, Indonesia. Int. J. Microbiol. 2022, 2022, 1–6. 10.1155/2022/9975333. PMC9532089 36204461

[ref25] Konicki W.; Aleksandrzak M.; Moszyński D.; Mijowska E. Adsorption of Anionic Azo-Dyes from Aqueous Solutions onto Graphene Oxide: Equilibrium, Kinetic and Thermodynamic Studies. J. Colloid Interface Sci. 2017, 496, 188–200. 10.1016/j.jcis.2017.02.031. 28232292

[ref26] Dey A. K.; Dey A.; Goswami R. Adsorption Characteristics of Methyl Red Dye by Na2CO3-Treated Jute Fibre Using Multi-Criteria Decision Making Approach. Appl. Water Sci. 2022, 12 (8), 179 10.1007/s13201-022-01700-9.

[ref27] Boubaker H.; Ben Arfi R.; Mougin K.; Vaulot C.; Hajjar S.; Kunneman P.; Schrodj G.; Ghorbal A. New Optimization Approach for Successive Cationic and Anionic Dyes Uptake Using Reed-Based Beads. J. Cleaner Prod. 2021, 307. 127218 10.1016/j.jclepro.2021.127218.

[ref28] Feszterová M.; Porubcová L.; Tirpáková A. The Monitoring of Selected Heavy Metals Content and Bioavailability in the Soil–Plant System and Its Impact on Sustainability in Agribusiness Food Chain. Sustainability 2021, 13 (13), 7021 10.3390/su13137021.

[ref29] Fakhar N.; Ayoub Khan S.; Ahmad Siddiqi W.; Alam Khan T. Ziziphus Jujube Waste-Derived Biomass as Cost-Effective Adsorbent for the Sequestration of Cd2+ from Aqueous Solution: Isotherm and Kinetics Studies. Environ. Nanotechnol., Monit. Manage. 2021, 16 (June), 100570 10.1016/j.enmm.2021.100570.

[ref30] Mwandira W.; Nakashima K.; Kawasaki S.; Arabelo A.; Banda K.; Nyambe I.; Chirwa M.; Ito M.; Sato T.; Igarashi T.; Nakata H.; Nakayama S.; Ishizuka M. Biosorption of Pb (II) and Zn (II) from Aqueous Solution by Oceanobacillus Profundus Isolated from an Abandoned Mine. Sci. Rep. 2020, 10 (1), 21189 10.1038/s41598-020-78187-4. 33273589 PMC7713119

[ref31] Madadgar S.; Doulati Ardejani F.; Boroumand Z.; Sadeghpour H.; Taherdangkoo R.; Butscher C. Biosorption of Aqueous Pb(II), Co(II), Cd(II) and Ni(II) Ions from Sungun Copper Mine Wastewater by Chrysopogon Zizanioides Root Powder. Minerals 2023, 13 ( (1), ). 106 10.3390/min13010106.

[ref32] Adetokun A. A.; Uba S.; Garba Z. N. Optimization of Adsorption of Metal Ions from a Ternary Aqueous Solution with Activated Carbon from Acacia Senegal (L.) Willd Pods Using Central Composite Design. J. King Saud Univ. - Sci. 2019, 31 (4), 1452–1462. 10.1016/j.jksus.2018.12.007.

[ref33] Aswini K.; Jaisankar V. Adsorption Treatment Of Heavy Metal Removal From Simulated Waste Water Using Rice Husk Activated Carbon (RHAC) And Its Polyvinylpyrrolidone (PVP) Composite As An Adsorbent. J. Water Environ. Sci. 2019, 3 (1), 460–470.

[ref34] Hong J.; Xie J.; Mirshahghassemi S.; Lead J. Metal (Cd, Cr, Ni, Pb) Removal from Environmentally Relevant Waters Using Polyvinylpyrrolidone-Coated Magnetite Nanoparticles. RSC Adv. 2020, 10 (6), 3266–3276. 10.1039/C9RA10104G. 35497719 PMC9048832

[ref35] Olawale S. A.; Bonilla-Petriciolet A.; Mendoza-Castillo D. I.; Okafor C. C.; Sellaoui L.; Badawi M. Thermodynamics and Mechanism of the Adsorption of Heavy Metal Ions on Keratin Biomasses for Wastewater Detoxification. Adsorpt. Sci. Technol. 2022, 2022, 1–13. 10.1155/2022/7384924.

[ref36] Candamano S.; Policicchio A.; Conte G.; Abarca R.; Algieri C.; Chakraborty S.; Curcio S.; Calabrò V.; Crea F.; Agostino R. G. Preparation of Foamed and Unfoamed Geopolymer/NaX Zeolite/Activated Carbon Composites for CO2 Adsorption. J. Cleaner Prod. 2022, 330 (June 2021), 129843 10.1016/j.jclepro.2021.129843.

[ref37] Qurrat-ul-Ain; Khurshid S.; Gul Z.; Khatoon J.; Shah M. R.; Hamid I.; Khan I. A. T.; Aslam F. Anionic Azo Dyes Removal from Water Using Amine-Functionalized Cobalt-Iron Oxide Nanoparticles: A Comparative Time-Dependent Study and Structural Optimization towards the Removal Mechanism. RSC Adv. 2020, 10 (2), 1021–1041. 10.1039/C9RA07686G. 35494463 PMC9048384

[ref38] Ma J.; Hou L.; Li P.; Zhang S.; Zheng X. Modified Fruit Pericarp as an Effective Biosorbent for Removing Azo Dye from Aqueous Solution: Study of Adsorption Properties and Mechanisms. Environ. Eng. Res. 2022, 27 (2), 200634 10.4491/eer.2020.634.

[ref39] Akpomie K. G.; Conradie J.; Adegoke K. A.; Oyedotun K. O.; Ighalo J. O.; Amaku J. F.; Olisah C.; Adeola A. O.; Iwuozor K. O. Adsorption Mechanism and Modeling of Radionuclides and Heavy Metals onto ZnO Nanoparticles: A Review. Appl. Water Sci. 2023, 13 (1), 20 10.1007/s13201-022-01827-9.

[ref40] Benzaoui T.; Selatnia A.; Djabali D. Adsorption of Copper (II) Ions from Aqueous Solution Using Bottom Ash of Expired Drugs Incineration. Adsorpt. Sci. Technol. 2018, 36 (1–2), 114–129. 10.1177/0263617416685099.

[ref41] Syafiuddin A.; Salmiati S.; Jonbi J.; Fulazzaky M. A. Application of the Kinetic and Isotherm Models for Better Understanding of the Behaviors of Silver Nanoparticles Adsorption onto Different Adsorbents. J. Environ. Manage. 2018, 218 (20), 59–70. 10.1016/j.jenvman.2018.03.066. 29665487

[ref42] Karimi-Maleh H.; Ayati A.; Davoodi R.; Tanhaei B.; Karimi F.; Malekmohammadi S.; Orooji Y.; Fu L.; Sillanpää M. Recent Advances in Using of Chitosan-Based Adsorbents for Removal of Pharmaceutical Contaminants: A Review. J. Cleaner Prod. 2021, 291. 125880 10.1016/j.jclepro.2021.125880.

[ref43] Sahin E. M.; Tongur T.; Ayranci E. Removal of Azo Dyes from Aqueous Solutions by Adsorption and Electrosorption as Monitored with In-Situ UV-Visible Spectroscopy. Sep. Sci. Technol. 2020, 55 (18), 3287–3298. 10.1080/01496395.2019.1676786.

[ref44] Zand A. D.; Abyaneh M. R. Adsorption of Lead, Manganese, and Copper onto Biochar in Landfill Leachate: Implication of Non-Linear Regression Analysis. Sustainable Environ. Res. 2020, 30 (1), 18 10.1186/s42834-020-00061-9.

[ref45] León G.; Gómez E.; Miguel B.; Hidalgo A. M.; Gómez M.; Murcia M. D.; Guzmán M. A. Feasibility of Adsorption Kinetic Models to Study Carrier-Mediated Transport of Heavy Metal Ions in Emulsion Liquid Membranes. Membranes 2022, 12 (1), 66 10.3390/membranes12010066. 35054592 PMC8778270

[ref46] Sharma R.; Raghav S.; Nair M.; Kumar D. Kinetics and Adsorption Studies of Mercury and Lead by Ceria Nanoparticles Entrapped in Tamarind Powder. ACS Omega 2018, 3 (11), 14606–14619. 10.1021/acsomega.8b01874. 30555981 PMC6289489

[ref47] Chakraborty R.; Asthana A.; Singh A. K.; Jain B.; Susan A. B. H. Adsorption of Heavy Metal Ions by Various Low-Cost Adsorbents: A Review. Int. J. Environ. Anal. Chem. 2022, 102 (2), 342–379. 10.1080/03067319.2020.1722811.

[ref48] Guo X.; Liu A.; Lu J.; Niu X.; Jiang M.; Ma Y.; Liu X.; Li M. Adsorption Mechanism of Hexavalent Chromium on Biochar: Kinetic, Thermodynamic, and Characterization Studies. ACS Omega 2020, 5 (42), 27323–27331. 10.1021/acsomega.0c03652. 33134695 PMC7594145

[ref49] Niknam S. M.; Kashaninejad M.; Escudero I.; Sanz M. T.; Beltrán S.; Benito J. M. Valorization of Olive Mill Solid Residue through Ultrasound-Assisted Extraction and Phenolics Recovery by Adsorption Process. J. Cleaner Prod. 2021, 316 ( (March), ). 128340 10.1016/j.jclepro.2021.128340.

[ref50] Bhadoria P.; Shrivastava M.; Khandelwal A.; Das R.; Langyan S.; Rohatgi B.; Singh R. Preparation of Modified Rice Straw-Based Bio-Adsorbents for the Improved Removal of Heavy Metals from Wastewater. Sustainable Chem. Pharm. 2022, 29 (May), 100742 10.1016/j.scp.2022.100742.

[ref51] Khandanlou R.; Ahmad M. B.; Masoumi H. R. F.; Shameli K.; Basri M.; Kalantari K. Rapid Adsorption of Copper(II) and Lead(II) by Rice Straw/Fe3O4 Nanocomposite: Optimization, Equilibrium Isotherms, and Adsorption Kinetics Study. PLoS One 2015, 10 (3), e0120264 10.1371/journal.pone.0120264. 25815470 PMC4376687

[ref52] Amer H.; El-Gendy A.; El-Haggar S. Removal of Lead (II) from Aqueous Solutions Using Rice Straw. Water Sci. Technol. 2017, 76 (5), 1011–1021. 10.2166/wst.2017.249. 28876243

[ref53] Zhang Y.; Zheng R.; Zhao J.; Ma F.; Zhang Y.; Meng Q. Characterization of H3PO4-Treated Rice Husk Adsorbent and Adsorption of Copper(II) from Aqueous Solution. Biomed Res. Int. 2014, 2014 (Ii), 1–8. 10.1155/2014/496878. PMC3942205 24678507

[ref54] Priya A. K.; Yogeshwaran V.; Rajendran S.; Hoang T. K. A.; Soto-Moscoso M.; Ghfar A. A.; Bathula C. Investigation of Mechanism of Heavy Metals (Cr6+, Pb2+& Zn2+) Adsorption from Aqueous Medium Using Rice Husk Ash: Kinetic and Thermodynamic Approach. Chemosphere 2022, 286 (P3), 131796 10.1016/j.chemosphere.2021.131796. 34391117

